# Cone-driven strong flash electroretinograms in healthy adults: Prevalence of negative waveforms

**DOI:** 10.1007/s10633-023-09957-4

**Published:** 2023-11-04

**Authors:** Xiaofan Jiang, Taha Bhatti, Ambreen Tariq, Shaun M. Leo, Nancy Aychoua, Andrew R. Webster, Pirro G. Hysi, Christopher J. Hammond, Omar A. Mahroo

**Affiliations:** 1 Institute of Ophthalmology, University College London, Bath Street, London, UK; 2 Department of Ophthalmology, King’s College London, St Thomas’ Hospital Campus, Westminster Bridge Road, London, UK; 3 Department of Twin Research and Genetic Epidemiology, King’s College London, St Thomas’ Hospital Campus, Westminster Bridge Road, London, UK; 4 NIHR Biomedical Research Centre at Moorfields Eye Hospital and the UCL Institute of Ophthalmology, 162 City Road, London, UK; 5 Physiology, Development and Neuroscience, University of Cambridge, Cambridge, UK; 6 Department of Translational Ophthalmology, Wills Eye Hospital, Philadelphia, PA USA

**Keywords:** Retina, Electroretinography, Retinal cone photoreceptors, Electrophysiology

## Abstract

**Purpose:**

Both rod and cone-driven signals contribute to the electroretinogram (ERG) elicited by a standard strong flash in the dark. Negative ERGs usually reflect inner retinal dysfunction. However, in diseases where rod photoreceptor function is selectively lost, a negative waveform might represent the response of the dark-adapted cone system. To investigate the dark-adapted cone-driven waveform in healthy individuals, we delivered flashes on a dim blue background, designed to saturate the rods, but minimally adapt the cones.

**Methods:**

ERGs were recorded, using conductive fibre electrodes, in adults from the TwinsUK cohort. Responses to 13 cd m^−2^ s white xenon flashes (similar to the standard DA 10 flash), delivered on a blue background, were analysed. Photopic and scotopic strengths of the background were 1.3 and 30 cd m^−2^, respectively; through a dilated pupil, this is expected to largely saturate the rods, but adapt the cones much less than the standard ISCEV background.

**Results:**

Mean (SD) participant age was 62.5 (11.3) years (93% female). ERGs from 203 right and 204 left eyes were included, with mean (SD) b/a ratios of 1.22 (0.28) and 1.18 (0.28), respectively (medians, 1.19 and 1.17). Proportions with negative waveforms were 23 and 26%, respectively. Right and left eye b/a ratios were strongly correlated (correlation coefficient 0.74, *p* < 0.0001). We found no significant correlation of b/a ratio with age.

**Conclusions:**

Over 20% of eyes showed b/a ratios less than 1, consistent with the notion that dark-adapted cone-driven responses to standard bright flashes can have negative waveforms. The majority had ratios greater than 1. Thus, whilst selective loss of rod function can yield a negative waveform (with reduced a-wave) in some, our findings also suggest that loss of rod function can occur without necessarily yielding a negative ERG. One potential limitation is possible mild cone system adaptation by the background.

## Introduction

The International Society for Clinical Electrophysiology of Vision (ISCEV) standard for full-field electroretinography specifies three flash strengths to be delivered in the dark, the strongest of which is termed the DA10 stimulus (corresponding to 10 photopic cd m^−2^ s) [1, 2]. Negative waveforms, in which the b-wave amplitude is selectively attenuated such that it is smaller than the a-wave amplitude, are not typically observed in healthy people in response to standard flash stimuli [3] and usually indicate loss of post-receptoral signals [4, 5]. However, in some conditions when rod photoreceptor responses are lost completely, the dark-adapted ERG represents a cone-driven response. In these cases, the a-wave is subnormal (due to loss of rod photoreceptor responses), but the b-wave can sometimes show a greater reduction, giving rise to a negative waveform [6]. It is thought that this might simply reflect the dark-adapted cone system response to the standard strong flash, which could have a negative waveform in many healthy individuals, but this can usually not be observed due to the simultaneous larger rod response [5–8].

We aimed to explore the form of the cone-driven response to such a flash strength in healthy individuals, specifically to investigate the prevalence of negative waveforms, and to compare the distribution of b-wave/a-wave amplitude ratios compared to the same flashes delivered in the dark. Such an investigation seeking to isolate the cone-driven component requires removal of the rod-driven component. The standard ISCEV light-adapting background (30 photopic cd m^−2^) does saturate the rods, but also significantly light-adapts the cone system, and so the response to flashes delivered on this background, whilst having no rod-driven components, cannot be taken to reflect the dark-adapted cone system response. A dim blue background can instead be used, such that the rods are largely saturated, but the cones are minimally desensitised. This has been used in a number of previous studies [9–15] to evaluate the dark-adapted cone system response.

Over 200 healthy adult volunteers from the TwinsUK cohort were previously recruited to undergo full-field ERG recordings in response to ISCEV standard stimuli and also to a range of experimental protocols [3, 16]. These included responses to white flashes of similar strength to the ISCEV standard strong flashes, but delivered in the presence of a dim blue rod-saturating background. We previously found that no participant showed a negative waveform in response to this flash strength when delivered in the dark [3]. The primary purpose of the present study was to investigate the response to such a flash when delivered in the presence of the blue rod-saturating background and to determine the proportion of these responses that showed a negative waveform (b/a ratio less than 1). Some of our findings have been presented in preliminary form (Association for Research in Vision and Ophthalmology Annual Meeting, 2021).

## Methods

### Participants

TwinsUK is a registry of largely healthy adult twins, who have volunteered to participate in research studies at St Thomas’ Hospital in London [17]. Participants were recruited from this cohort and gave informed consent. Participants were also recruited from the research team for control experiments and gave informed consent. Recordings were also made in a patient with genetically confirmed achromatopsia (no functioning cones) and analysed as an additional control. The study had local research ethics committee approval and complied with the tenets of the Declaration of Helsinki.

### Procedures

The Diagnosys ColorDome with Espion software (Diagnosys, Lowell, MA) was used for stimuli and recording. Filter settings for recordings were as set by the manufacturer (high pass 0.312 Hz, low pass 300 Hz). Responses were recorded simultaneously from both eyes with conductive fibre electrodes placed in the inferior conjunctival fornices. Consistency of position was checked during and after recordings. Indifferent skin electrodes were placed on the temples and a ground electrode on the forehead.

TwinsUK participants underwent pharmacological mydriasis and 20-min dark adaptation prior to commencement of stimuli. They were then exposed to the standard ISCEV dark-adapted stimuli (DA 0.01, DA 3 and DA 10, delivered using LEDs), followed by additional white xenon flashes that were first delivered in the dark and then in the presence of the blue LED background (1.3 photopic and 30 scotopic cd m^−2^). The peak wavelength of the blue LED was 445 nm (half-bandwidth 20 nm). The retinal illuminance of such a background, assuming a pupil diameter of 8 mm, would be 65.3 photopic and 1508 scotopic trolands. For a pupil diameter of 7 mm, the expected illuminances would be 50.0 photopic and 1155 scotopic trolands. This is in excess of the 1000 scotopic trolands at which rod saturation is felt to be largely complete. Inter-stimulus intervals ranged from 0.5 s for the weaker stimuli to 20 s for the stronger flashes. The xenon flashes included a stimulus that was similar in strength to the standard DA10 strong flash. The nominal flash strength was 10 cd m^−2^ s, but when measured independently using a photometer with photopic filter (and confirmed by subsequent calibration by the manufacturer), the flash strength was closer to 13 cd m^−2^ s. The corresponding strength in scotopic units as given by the Espion software was 21 cd m^−2^ s.

### Analysis

In this study, ERGs recorded to the 13 cd m^−2^ s flashes delivered in the dim blue background were analysed. Those responses containing excessive noise, drift or artefacts (for example, due to blinking) were removed using criteria described in previous studies [3, 10, 16]. Typically, less than 10% of the responses were removed, and in many cases, no responses needed to be removed. Remaining responses were averaged. A-wave and b-wave amplitudes were extracted from these responses. The proportion of responses with a negative waveform (b/a ratio less than 1) was calculated. In addition, the responses to similar strength flashes delivered in the dark obtained from the same cohort [3] were analysed, so that the distributions of b/a ratios of those responses could be compared with those recorded on the blue background.

### Additional experiments

Some control experiments were performed as follows: responses to the same flash strength delivered on the blue background and on the ISCEV standard light-adapting background were recorded in the same individual; ERGs to the same and additional flash strengths were recorded from two individuals in the presence of the blue background and additional backgrounds that were photopically (but not scotopically) matched. For the red LED background, the peak wavelength was 630 nm (half-bandwidth 20 nm). These waveforms will be presented first in the Results section, together with ERGs in a patient with achromatopsia (in response to the strong flash delivered in the dark and on the blue background), prior to presenting the findings of the main study.

## Results

### Comparison of waveforms on different backgrounds (control experiments)

**Fig. 1:**
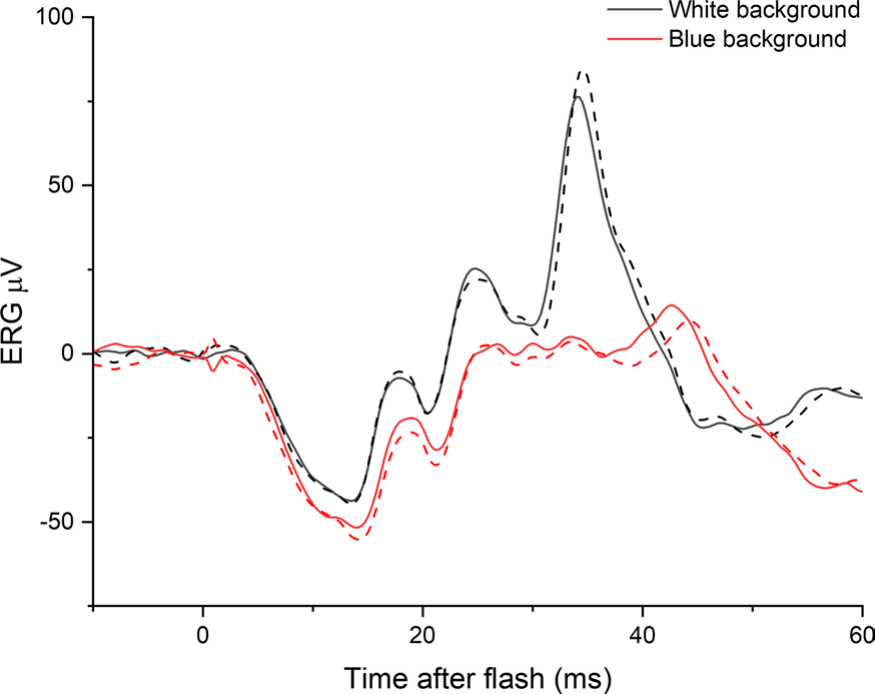
Responses to 10 cd m^−2^ s flashes delivered in the presence of two backgrounds, recorded from the same individual. Solid traces are averaged from the right eye; dashed traces are from the left eye. Black traces are responses to flashes delivered in the presence of the ISCEV standard light-adapting white background (30 photopic cd m^−2^, following 10-min adaptation to this background); red traces are responses to flashes delivered in the presence of a dim blue rod-saturating background (1.3 photopic cd m^−2^). Scotopic strengths of the two backgrounds were 86 and 30 scotopic cd m^−2^, respectively

Figure 1 shows responses to strong white flashes (10 cd m^−2^ s, the strength of the standard DA10 stimulus), recorded in the same individual, in the presence of the ISCEV standard light-adapting white background (30 photopic cd m^−2^; 86 scotopic cd m^−2^) or in the presence of the blue background used in the present study (1.3 photopic cd m^−2^; 30 scotopic cd m^−2^). The form of the response is quite different in the two backgrounds, with a lower b/a ratio clearly evident on the blue background compared with the white background. It is likely that the response obtained on the blue background is closer to the dark-adapted cone system response, given that the blue background is much weaker in photopic terms.

**Fig. 2:**
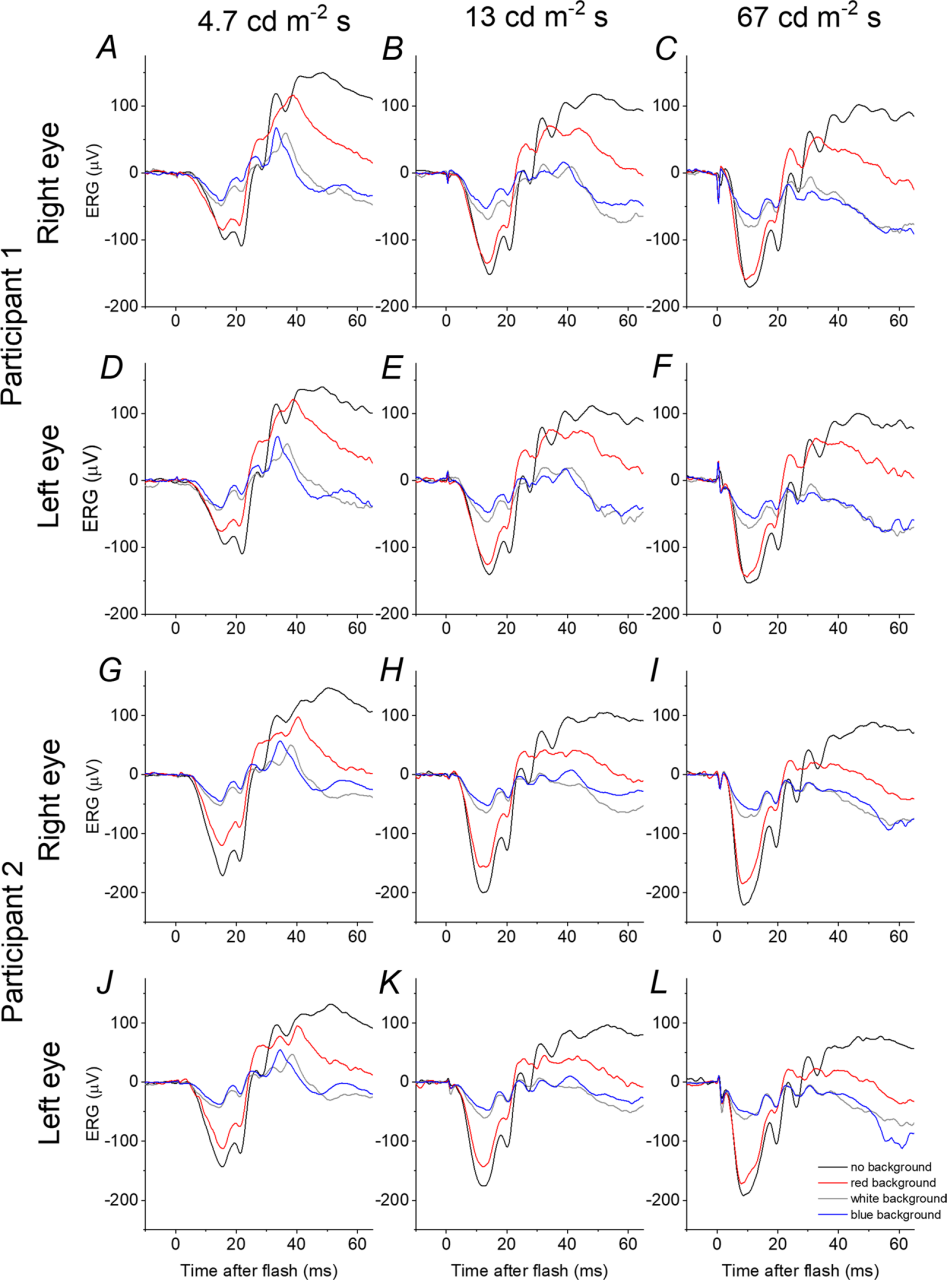
Responses to white flashes of three different flash strengths (4.7, 13 and 67 photopic cd m^−2^ s) recorded in the dark and on three photopically matched backgrounds (photopic luminance 1.3 cd m^−2^) in two participants. The scotopic luminances of the red, white and blue backgrounds were 0.11, 3.7 and 30 scotopic cd m^−2^, respectively. Pupil diameter for each of the participants was 8 mm. Traces are averages of multiple flash presentations. **A**–**F** Responses from the right eye (**A**–**C**) and left eye (**D**–**F**) of Participant 1 (a 26-year-old healthy female). **G**–**L** Responses from the right eye (**G**–**I**) and left eye (**J**–**L**) of Participant 2 (a 32-year-old healthy male)

In Fig. 2, results of another control experiment are presented. The traces plot ERGs elicited by three strengths of white flash (4.7, 13 and 67 photopic cd m^−2^ s) in two individuals in the dark (black traces) and in the presence of three different backgrounds that were matched to the same photopic luminance (1.3 photopic cd m^−2^), but different scotopic luminances (by varying the wavelength spectrum of the backgrounds). The backgrounds are red (0.11 scotopic cd m^−2^), white (3.7 scotopic cd m^−2^) and blue (30 scotopic cd m^−2^), and the corresponding ERGs are plotted as red, grey and blue traces, respectively. In all cases, the responses in the dark are the largest, as expected, given that they contain large rod and cone system contributions. The red traces show amplitudes lower, but not much lower, than those obtained in the dark. This is also expected: the scotopic strength of the background is low, and so there will be some small degree of stimulation (and desensitisation) of both rod and cone systems.

The white traces show a substantial reduction, compared with the red traces. Here, the photopic strengths are again similar, but the scotopic strength of the background is substantial (delivering an estimated retinal illuminance of 186 trolands through an 8-mm-diameter pupil, more than 30 times the strength of the red background). The rods will be substantially desensitised by this background [18]. The blue traces are similar to the grey traces, but consistently of lower amplitude (evident in both eyes of both subjects for all flash strengths), and this is particularly seen for the strongest flash. Given the photopic strength is again similar, this is consistent with further saturation of the rods (removal of a residual rod component). The background luminance is now expected to near fully saturate the rod circulating current [18].

### Recordings from patient with achromatopsia

**Fig. 3:**
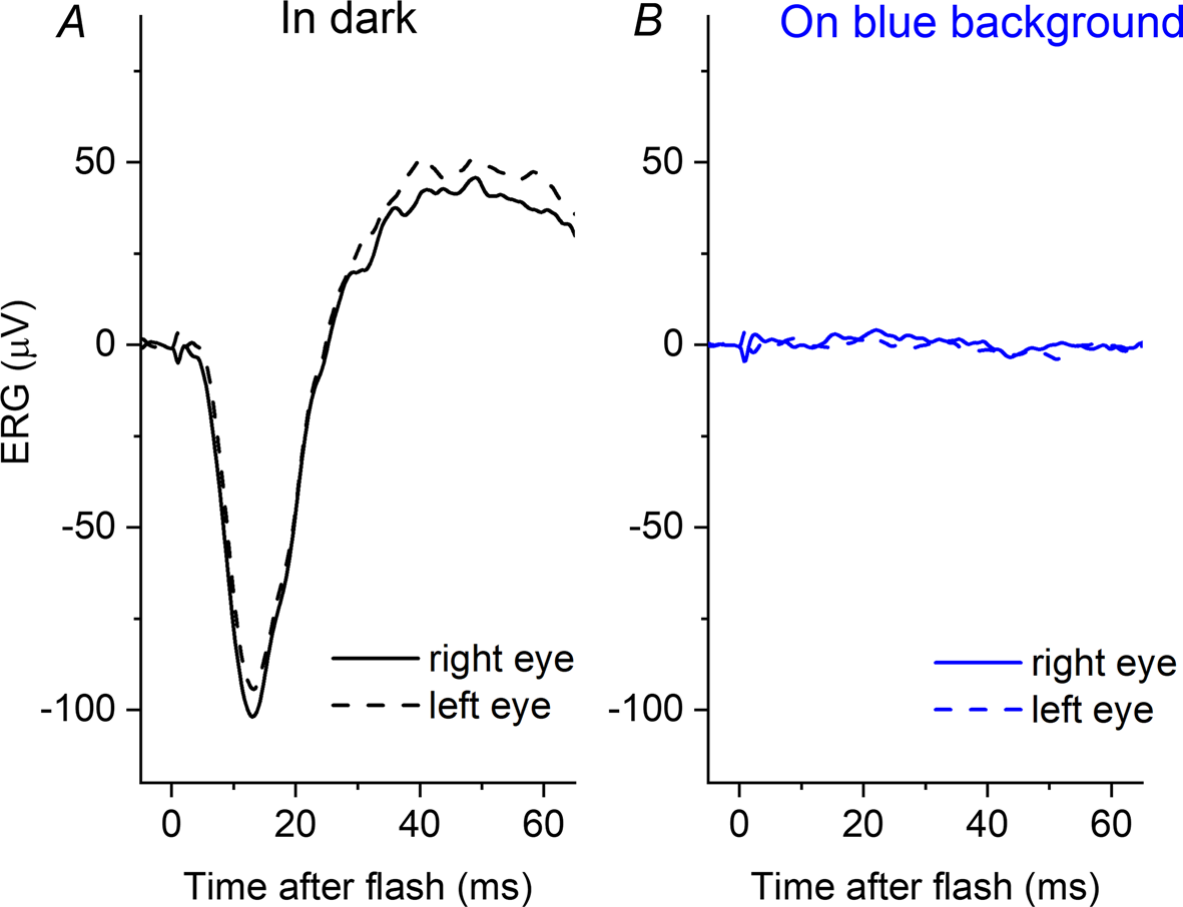
Responses to the white xenon flash (13 cd m^−2^ s) recorded in a 20-year-old patient with genetically proven achromatopsia. Responses are averages of multiple flash presentations. **A** Response recorded in the dark. **B** Response to the same flash delivered in the presence of the blue rod-saturating background (1.3 photopic cd m^−2^; 30 scotopic cd m^−2^). The ERG on the blue background is largely undetectable, consistent with the notion that this background has largely saturated the rods

Figure 3 shows responses from a patient with genetically confirmed achromatopsia (bi-allelic variants in *CNGA3*, encoding the alpha subunit of the cyclic nucleotide-gated cation channel in cone outer segments). This patient only has rod-driven responses, with no functioning cones. The left panel shows responses to the strong flash delivered in the dark; the right panel shows responses to the same flash delivered on the blue background. The latter response is not distinguishable from noise, confirming that this background is sufficient to saturate the rods to the extent that a rod-driven ERG response to a flash of this strength is undetectable.

### Results from the full cohort (main study)

**Fig. 4:**
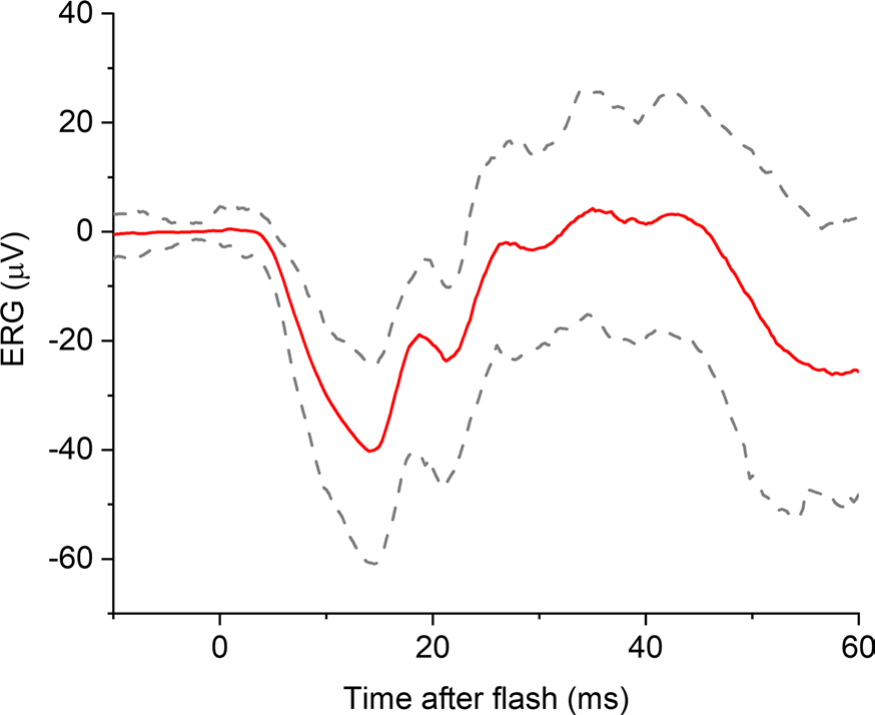
Averaged ERG response to 13 cd m^−2^ s white flash delivered on the blue background (averaged from all twin participants). Solid red trace shows the waveform averaged from all participants. Dashed grey traces denote 2.5th and 97.5th percentiles at each time point

Strong flash ERGs were recorded in 211 TwinsUK participants. Mean (SD) age was 62.5 (11.3) years, and 93% were female (reflecting the demographics of the TwinsUK cohort). After exclusion of traces contaminated by noise artefact, responses from 203 right and 204 left eyes were included. Figure 4 depicts the response averaged from all participants (with the dashed lines showing the 2.5th and 97.5th centile amplitudes at each time point). The mean (SD) b/a ratio was 1.22 (0.28) and 1.18 (0.28) for right and left eyes, respectively. Median values were 1.19 and 1.17, respectively. No significant correlations with age were found for the b/a ratio.

**Fig. 5:**
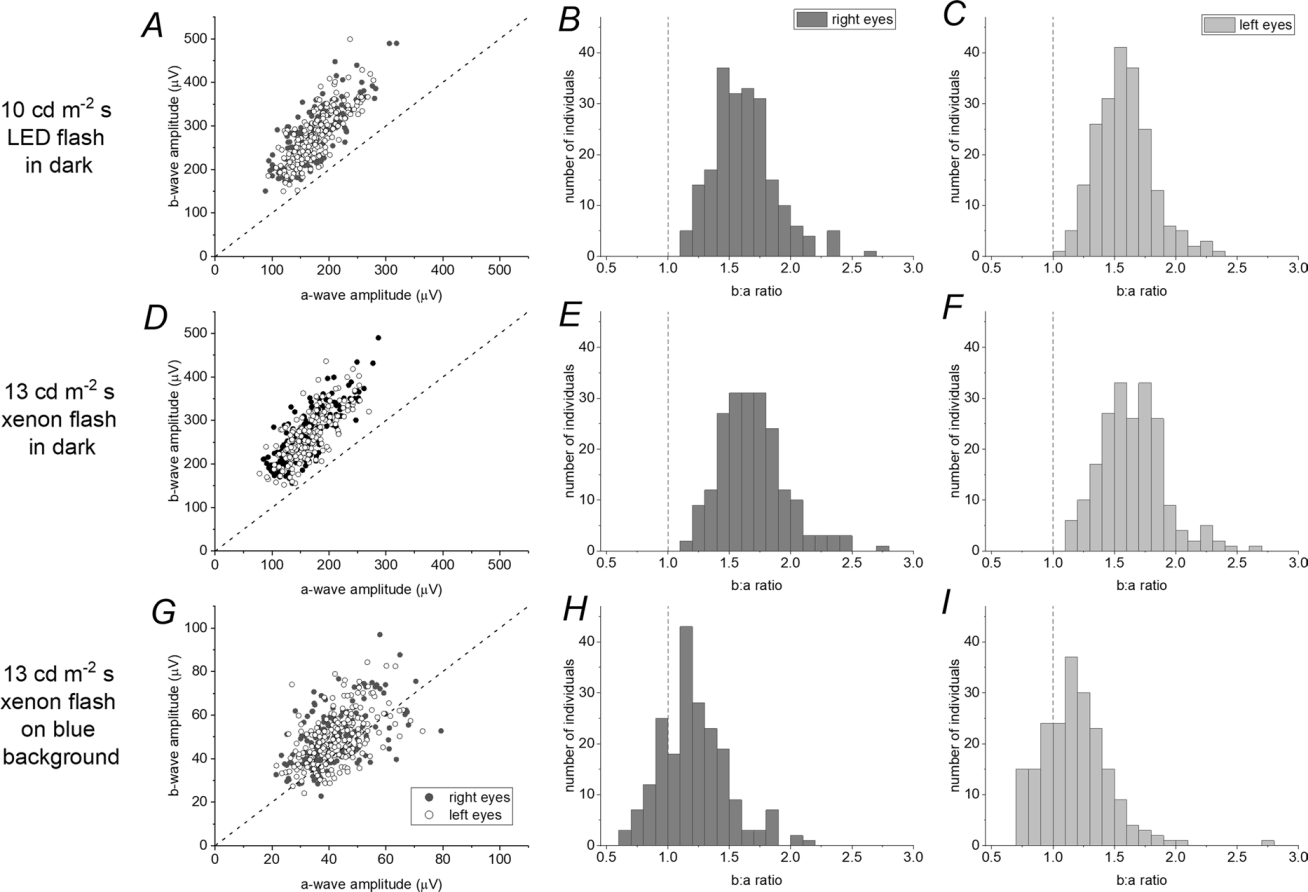
Comparison of b/a ratios in the dark and on the blue background. Left panels plot b-wave amplitudes against a-wave amplitudes. The dashed 45-degree line denotes a b/a ratio of 1. The middle column and right-hand panels show distributions of b/a ratios for right and left eyes, respectively. **A**–**C** Data for the standard ISCEV DA10 flash delivered in the dark. Mean (SD) a-wave amplitudes were 172 (42) and 175 (36) microvolts for right and left eyes, respectively; mean (SD) b-wave amplitudes were 274 (62) and 274 (59) microvolts, respectively; mean (SD) b/a ratios were 1.62 (0.25) and 1.58 (0.23), respectively. **D**–**F** Data for the white xenon 13 cd m^−2^ s flash (focus of the present study) delivered in the dark. Mean (SD) a-wave amplitudes were 161 (41) and 163 (37) microvolts for right and left eyes, respectively; mean (SD) b-wave amplitudes were 267 (60) and 265 (56) microvolts, respectively; mean (SD) b/a ratios were 1.69 (0.27) and 1.64 (0.26), respectively. The amplitudes and ratios are very similar to those shown in **A**–**C** as expected. **G**–**I** Data for the white xenon 13 cd m^−2^ s flash delivered in the presence of the blue background. Mean (SD) a-wave amplitudes were 41.9 (10.1) and 42.6 (9.2) microvolts for right and left eyes, respectively; mean (SD) b-wave amplitudes were 49.7 (12.2) and 49.4 (11.9) microvolts, respectively; mean (SD) b/a ratios were 1.22 (0.28) and 1.18 (0.28), respectively. The b/a ratio distribution is clearly shifted towards lower ratios compared to the distribution in the dark. More than 20% of the b/a ratios are below 1

**Fig. 6:**
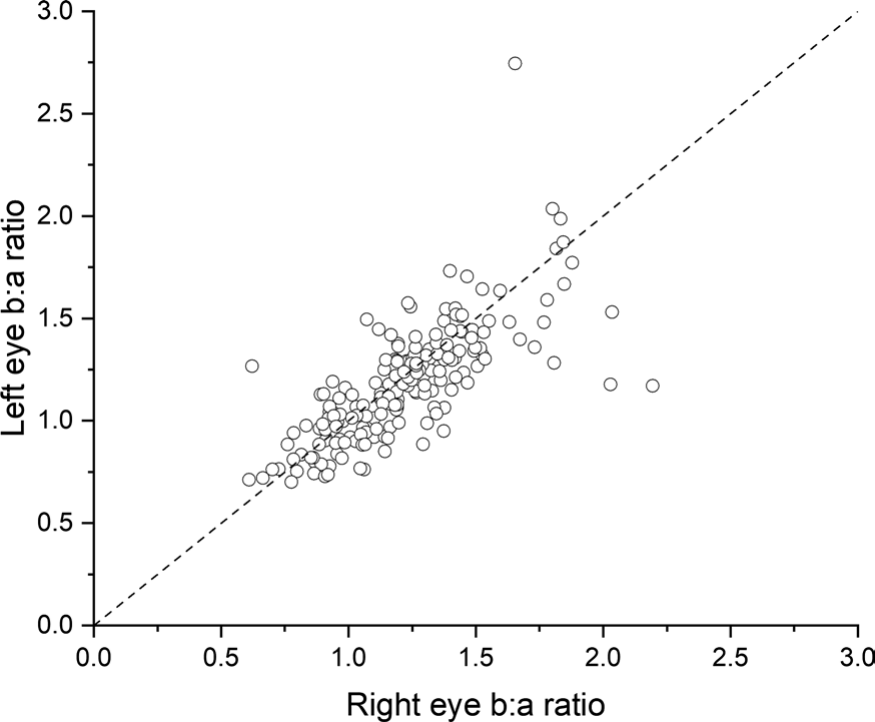
Scatter plot of left eye b/a ratio against right eye b/a ratio for ERGs elicited by the white xenon flash (13 photopic cd m^−2^ s) delivered on the blue background. The dashed line denotes identical ratios for the two eyes

In Fig. 5, the distributions of the b/a ratios are examined (with mean a-wave and b-wave amplitudes given in the legend). Here, comparison is made to the distribution of b/a ratios obtained in the dark. The left-hand panels in each row plot b-wave amplitudes against a-wave amplitudes. The 45-degree line denotes a b/a ratio of 1. The middle and right-hand panels of each row depict the distributions of b/a ratios. The top panels plot data relating to the ISCEV standard DA10 flash delivered in the dark, as reported in our recent study [3]. The middle panels plot corresponding data for the 13 cd m^−2^ s white xenon flash investigated in the current study, but delivered in the dark. These data are very similar to the standard DA10, as would be expected. The lowermost panels plot the corresponding data for the same flash, but delivered on the blue background. The distribution of b/a ratios is quite different (clearly shifted to lower values for the flash delivered on the blue background). In the top and middle panels, no ratios were less than 1; for the lowest panels, in contrast, a significant proportion was below 1: 47 right eyes and 53 left eyes had b/a ratios less than 1, corresponding to 23 and 26%, respectively. Finally, right and left eye b/a ratios for this stimulus are compared in Fig. 6. These were highly correlated, with a Pearson correlation coefficient of 0.74 (*p* < 0.0001).

## Discussion

In this study, we analysed responses to flashes of similar strength to the ISCEV standard strong flash (DA10), but delivered in the presence of a dim blue rod-saturating background, aiming to investigate the form of the cone-driven response, specifically to quantify the proportion with b/a ratio less than 1. We found that over 20% showed negative waveforms. We anticipate that these responses will be closer to the dark-adapted cone system response than responses recorded on the standard ISCEV photopic background. Thus, in patients with complete, or near complete, loss of rod function, a negative ERG (with reduced a-wave) could reflect the normal dark-adapted cone system response. Our study additionally suggests that it might also be possible for complete loss of rod function to occur without a negative ERG.

Comparing directly with the b/a ratio distribution for the same flash delivered in the dark (Fig. 5), the b/a ratios are clearly smaller on the blue background, consistent with the isolated dark-adapted cone system response to this flash strength having a lower b/a ratio. Figure 5 also confirms that the response to this flash delivered in the dark is similar to that elicited by the ISCEV standard 10 cd m^−2^ s flash. Thus, although the flash strengths are slightly different, and the sources of the white flash differ (xenon or LED), the a-wave and b-wave amplitudes, as well as their ratios, are similar for the two stimuli.

In our control experiments (Fig. 2), we confirmed that the scotopic strength of the background was likely sufficient to substantially remove the rod contribution. If the same photopic background strength was used, but with minimal scotopic luminance (i.e. a red background), responses were reduced compared to those in the dark, but not substantially. With the same photopic luminance, but increasing the scotopic background luminance further (using a white or blue background), the responses were reduced more markedly, with a clear reduction in b/a ratio. The differences between the ERGs elicited on the white and blue backgrounds were not large, but were clearly evident. This would be expected, as the scotopic luminance of the white background is likely to be sufficient to saturate > 70% of the rod photoreceptor circulating current [18]. The additional increase in luminance moving from the white to the blue background would be likely to further, nearly fully, saturate the rod photoreceptor current.

**Fig. 7:**
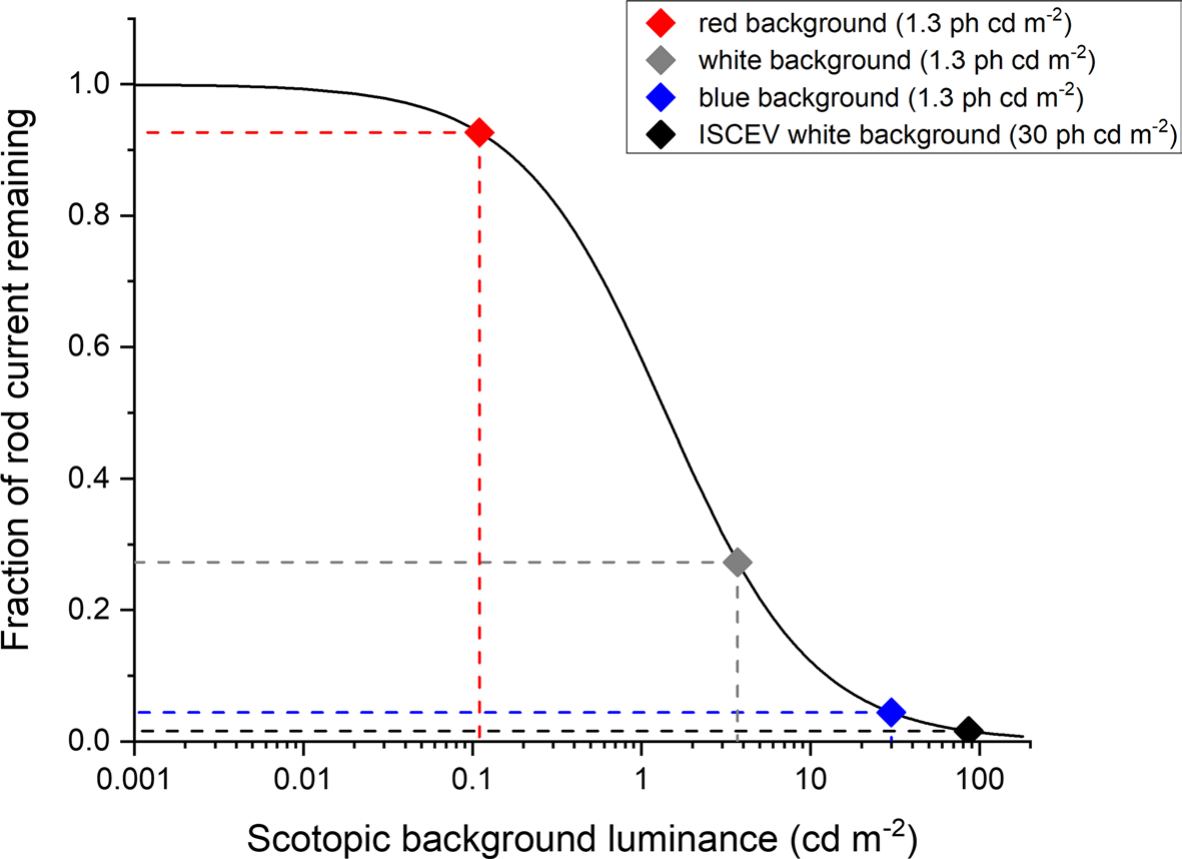
Theoretical curve showing suppression of rod circulating current as steady background luminance increases. This curve plots the hyperbolic function from Thomas and Lamb [18], where fraction current remaining is given by I0/(IB + I0) where IB is the background strength and I0 is the background strength that suppresses the current by 50%. In this figure, I0 has been set at 1.4 cd m^−2^, which corresponds to 70 Td (the mean for their subjects) for a pupil diameter of 8 mm. The points highlighted correspond to the different backgrounds in Fig. 2 as well as an additional point corresponding to the ISCEV standard white background

Thomas and Lamb investigated suppression of human rod circulating current at different background strengths using the ERG a-wave elicited by very strong flashes [18]. They found that the estimated fractional current followed a hyperbolic function, when plotted against background illuminance, with a mean half-saturating retinal background illuminance of 70 Td. The function they derived is replotted in Fig. 7. The *x*-axis plots background luminance in scotopic cd m^−2^ (assuming an 8 mm diameter pupil, which was the measured dilated pupil diameter in our control participants). Points corresponding to the backgrounds used in Fig. 2 have been highlighted. The red background suppresses very little (< 10%) current; the white background (1.3 photopic cd m^−2^; 3.7 scotopic cd m^−2^) is expected to suppress ~ 73% of the rod dark current; the blue background is expected to suppress ~ 96%. This is consistent with the findings shown in Fig. 2, where the white background is associated with a substantially reduced flash response, and the blue background reduces the response further by a small additional amount.

**Fig. 8:**
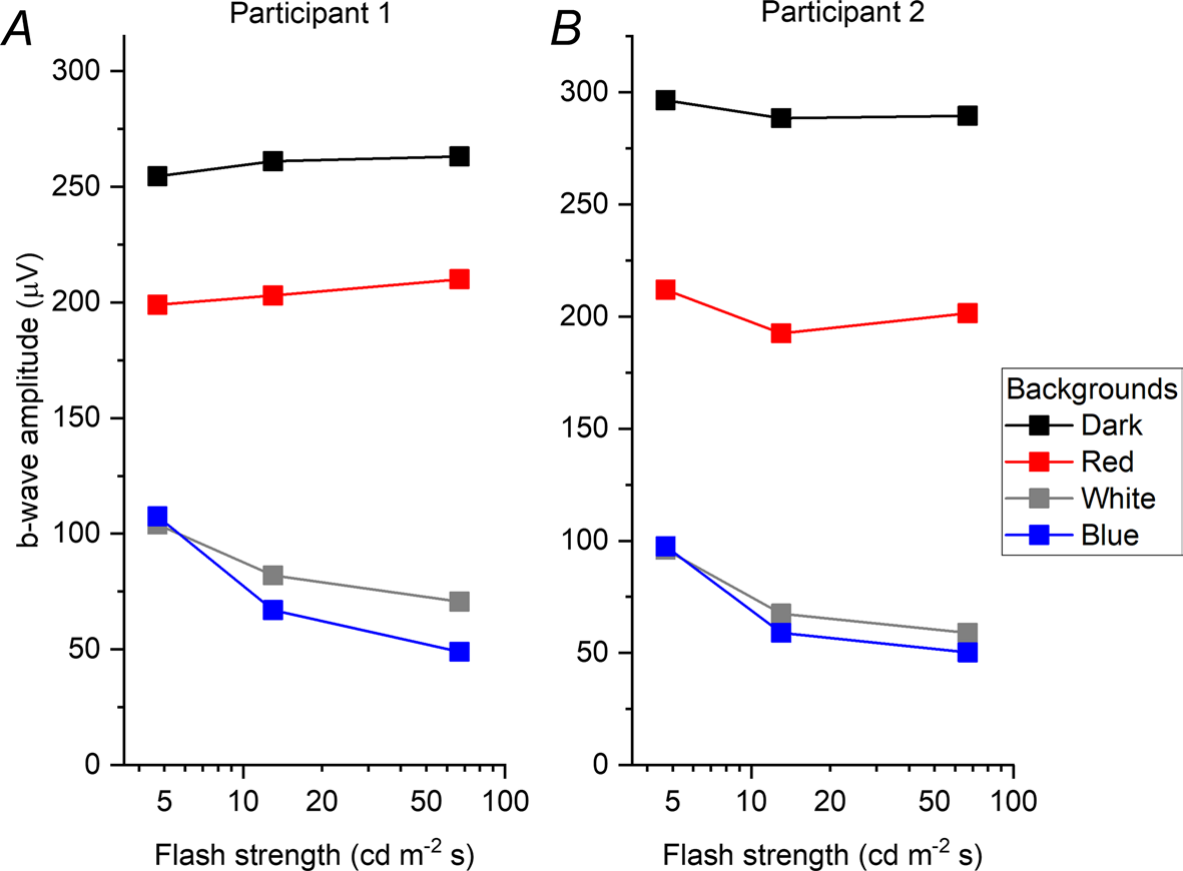
B-wave amplitudes plotted against flash strength for the different backgrounds in Fig. 2. Amplitudes have been averaged from right and left eyes. In both participants, a clear reduction in b-wave amplitude with increasing flash strength is seen for the white and blue backgrounds (which remove much, or nearly all, of the rod system contribution, respectively), which is consistent with the descending limb of the photopic hill phenomenon. **A** Data for Participant 1. **B** Data for Participant 2

A well-described feature of cone system responses is the “photopic hill” phenomenon, where, above a particular flash strength, the b-wave amplitude starts to decrease with increasing flash strength [19, 20]. Figure 8 plots the b-wave amplitudes of the traces shown in Fig. 2. For the traces in the dark and on the red background, the b-wave amplitudes appear not to change much with flash strength. However, for traces on the white and blue backgrounds, a clear reduction in b-wave as flash strength increases is evident, indicating that the two stronger flashes, 13 and 67 cd m^−2^ s would be beyond the peak of the photopic hill, representing the descending limb. This is consistent with the “photopic hill” phenomenon also applying to the dark-adapted cone system (not solely to the light-adapted cone system), becoming apparent when the rod system contributions to the responses have been largely removed (by the white and blue backgrounds). This also makes it likely that the dark-adapted cone system response to flashes stronger than the standard DA10 flash will have an even lower b/a ratio, as they will be further along the descending limb of the photopic hill. The flash strength that was the focus of the present study was chosen as it corresponds to the standard strong flash; strictly speaking, the findings are thus applicable to this luminance and cannot necessarily be generalised to other stimulus strengths.

Methods that can be used in healthy individuals to record the dark-adapted cone system response (without a rod contribution) include delivering red flashes (which stimulate the L-cones and M-cones more than the rods, although stronger red flashes will contain a rod-driven contribution), delivering flashes within 1–2 s following a very strong rod-saturating bright flash (the cones will have recovered by this time whilst the rods remain in saturation), and delivering flashes in the presence of a steady background that is sufficiently bright to keep the rods in saturation [21]. Each method has specific limitations, but delivering flashes in the presence of a background is more comfortable for participants and less time-consuming. Furthermore, our recordings from the patient with achromatopsia (showing no clear detectable ERG to the flash delivered in the presence of the blue background; Fig. 3) support the notion that for the particular flash strength and background employed in this study, any rod-driven response is largely removed.

Although an extended protocol exists, specifying delivery of a dim red flash in the dark-adapted state to probe the dark-adapted cone system [22], the response to such a stimulus includes a substantial rod-driven component. One advantage of the method of employing a dim blue background is that rod-driven components are largely removed, and one can probe the cone system response to brighter flashes, in health and disease, permitting estimates of the cone system contribution to dark-adapted responses to stronger flashes.

Verdon et al. [21] compared the different methods of cone system isolation and found that the response on a steady background was less reflective of the dark-adapted cone response, compared with other methods. However, that study employed the standard ISCEV white background, which significantly light-adapts the cone system. In the present study, a dimmer blue background was used, which would be expected to cause less cone system light adaptation (and Fig. 1 shows the difference between flash responses on the two backgrounds). Nevertheless, we cannot exclude some degree of adaptation of the cone system, and hence, our estimate of the proportion of individuals with a negative dark-adapted cone system response could be an underestimate. Future studies could be conducted in patients with known loss of rod-driven responses (including patients with fundus albipunctatus, vitamin A deficiency or Oguchi disease) to investigate whether responses in the dark and in the presence of the blue background used in the present study show similar waveforms. Any differences seen might be attributable to residual rod responses in the dark or to some adaptation of the cone system by the blue background.

Another factor to consider is that even with the rods largely saturated such that they are not expected to generate a detectable response to the flashes delivered, they will be hyperpolarised in the presence of the background, whilst in the dark, they are depolarised. As lateral rod-cone system interactions exist in the retina, it is not known for certain that the cone system response would be identical in the two situations.

Other limitations of our study include the specific demographics of our cohort, which could limit generalisability of our findings to other populations. Although all participants underwent mydriasis, pupil areas were not specifically measured, and lens opacity was not specifically graded. It is possible that differences in dilated pupil area and media opacity will affect retinal illuminance and give rise to some of the variability seen in b/a ratios. However, this applies to ERG recording generally and so does not represent a weakness of the study, since the aim was to examine the distribution in a general adult population. Smaller pupil diameters, however, will lower the retinal illuminance of the blue background (and hence the degree of rod saturation).

The electronegative ERG response to strong flashes in the dark-adapted state is a useful clinical sign [4, 5]; it is defined by a normal amplitude a-wave with a subnormal b-wave (that is smaller in amplitude than the a-wave) and indicates a site of impairment in the visual pathway that is after rod phototransduction, often narrowing the differential diagnosis considerably. Our findings support the notion that when rod photoresponses are selectively abolished (and the strong flash a-wave is hence markedly subnormal), an intact cone system can give rise to a negative waveform in some individuals, and the negative waveform should not then be taken to necessarily reflect post-phototransduction impairment.

## Declarations

### Statement on the welfare of animals

No animal experiments were done, so there is no reference to welfare of animals.
